# Retrospective assessment of the frequency of cancer in the population of kidney transplant recipients - the experience of two transplant centers

**DOI:** 10.3389/fonc.2025.1497691

**Published:** 2025-07-14

**Authors:** Karolina Komorowska-Jagielska, Alicja Dębska-Ślizień, Aureliusz Kolonko, Zbigniew Heleniak, Jakub Ruszkowski, Kamila Czarnacka, Beata Imko-Walczuk, Bogdan Biedunkiewicz, Barbara Bułło-Piontecka, Beata Bzoma, Andrzej Chamienia, Justyna Gołębiewska, Joanna Konopa, Ewa Król, Monika Lichodziejewska-Niemierko, Przemysław Rutkowski, Agnieszka Tarasewicz, Andrzej Więcek, Sławomir Lizakowski

**Affiliations:** ^1^ Department of Nephrology, Transplantology and Internal Medicine, Medical University of Gdańsk, Gdańsk, Poland; ^2^ Department of Nephrology, Transplantology and Internal Medicine, Medical University of Silesia, Katowice, Poland; ^3^ Dialysis center “Fresenius Nephrocare Poland”, Ostróda, Poland; ^4^ Skin and Venereology Clinic, Copernicus Medical Entity, Gdańsk, Poland; ^5^ Department of Palliative Medicine, Medical University of Gdańsk, Gdańsk, Poland; ^6^ Department of Internal Medicine and Pediatric Nursing, Faculty of Health Sciences, Medical University of Gdańsk, Gdańsk, Poland

**Keywords:** kidney transplant, malignacies, cancer, kidney recipients, oncology in transplantation

## Abstract

**Introduction:**

Cancer is one of the main causes of death among kidney recipients. The risk of cancer in kidney transplant recipients (KTRs) is 2–3 times higher as compared to the general population.

**Aim:**

Retrospective assessment of the occurrence of cancer in the population of KTRs – based on data from two transplant centers.

**Material:**

The study included a total of 246 KTRs, transplanted between 1980 and 2021, who were diagnosed with malignancy (the study did not include patients whose only cancer was non-melanoma skin cancer; NMSC).

**Results:**

261 malignant tumors were diagnosed in 246 KTRs, 3 tumor was a recurrence, and the rest occurred *de novo*. The most common cancers in women were breast cancers (17.8%), colon cancers (14.5%), lung cancers and post-transplant lymphoproliferative disorder (PTLD) (8.9% each). In males, the most common cancers were native kidney cancer (16.4%), lung cancer (15.7%) and prostate cancer (14%). During the study period, among KTRs who developed solid organ malignancy, NMSC was diagnosed in 7.3% of recipients. The average time of occurrence of malignant tumors was 84.5 months/7 years after kidney transplantation (KTx), and most cancers developed in the range of 1–5 years (33.6%) and 5–10 years (34.42%) after KTx. Nearly half (48.8%) of patients died due to cancer.

**Conclusions:**

Similarly to the general population, the most common cancers among KTRs included breast and prostate cancer, as well as colorectal and lung cancer. Attention should be paid to the extremely frequent occurrence of native kidney and lymphatic system cancers in this group of KTRs. The frequent occurrence of cancer in KTRs requires systematic screening in this population.

## Introduction

Epidemiological data from 40 countries in Europe reveal the incidence of 4 million new cases of solid-organ cancer (excluding non-melanoma skin cancer; NMSC) and 1.9 million cancer-related deaths in 2020. The most common were as follows: breast in females, prostate cancer in males, colon cancer, and lung cancer in both sexes, collectively accounting for half of all cancer cases (1).

In Poland, cancer is the second leading cause of death in the general population (2–4), causing 21.8% of male deaths and 20% of female deaths in 2020 (4). They constitute a significant health problem, especially among young and middle-aged people (25–64 years old). While the incidence of malignant tumors remains stable among males, it is steadily increasing among females (3). In the female population for several years cancer has been the most common cause of death before the age of 65, accounting for 28.3% of deaths among young women and 41.6% of deaths among middle-aged women (4). Lung cancer is the predominant cancer in both sexes, accounting for one-third of all cancer-related deaths among males (3).

KTRs have a significantly higher risk of the cancer development than the general population.

This is due to the use of immunosuppressive therapy after KTX which reduces immunosurveillance against tumoral cells and promote infections with oncogenic viruses leading to an increased cancer risk.

In addition, any treatment for rejection of the transplanted organ, multidrug IS regimens or IS therapy before KTx (treatment of primary kidney disease) increases the overall “burden” of IS, which is of significant importance. KTRs are also distinguished by the fact that cysts in their own kidneys, acquired or formed in the course of ADPKD, can undergo malignant transformation over time. In addition, impaired kidney function is a significant factor increasing the risk of developing kidney cancer due to more severe oxidative stress in this population (5). As a consequence, this causes a “transfer” of cancer risk (especially of the urinary system) from the period of ESRD to the period after transplantation (Tx). All this means that kidney recipients are developing cancer at a younger age. On the other hand, standard risk factors for cancer, such as smoking, obesity, alcohol consumption, excessive exposure to ultraviolet radiation, advanced age, occupational exposure to carcinogens, or genetic predisposition, are present in the KTRs population, which, together with kidney transplant-specific factors, significantly increase the risk of cancer in this population.

Cancer is one of the three main causes of death among kidney transplant recipients (KTRs) (6–11) and thus, cancer after kidney transplantation (KTx) poses a significant challenge for clinicians.

This study aimed to retrospectively assess the occurrence of cancer within the KTRs based on data from two transplant centers.

## Materials and methods

The inclusion criteria consisted of the KTRs diagnosed with malignant neoplasia, including melanoma. The exclusion criteria included the KTRs with only NMSC and diagnosis of neoplasm after the patient’s death (in the autopsy procedure).

The group of patients with NMSC was excluded because it is a group of tumors that are characterized by a different course and different pathophysiology in comparison with solid organ tumors after Tx. In a view of the above, a separate patient database is devoted to NMSC. Only one case of a recipient in whom cancer was diagnosed in postmortem examination was excluded because it was impossible to calculate the survival time from diagnosis to death, which was of interest to us.

Moreover, this cohort excluded patients with incomplete medical documentation and patients moved to another transplant center.

All KTRs who were transplanted between 1980 and 2021 in either of the two transplant centers (TC) in Poland: the Department of Nephrology, Transplantology and Internal Medicine, Medical University of Gdansk, in Gdansk and the Departmentof Nephrology, Transplantology and Internal Medicine, Medical University of Silesia in Katowic were screened towards inclusion/exclusion criteria of our study.

Cancers with a common location and similar prognosis were analyzed together (e.g. head&neck, PTLD (regardless of immunophenotype).

KTRs with cancer diagnosis without disease recurrence 5 years after treatment were considered cured.

### Statistical analysis

Microsoft Office Excel 365 and rStudio 2023.06.0 (R 4.2.3 and packages: dplyr 1.1.3, readxl 1.4.3, gtsummary 1.7.2) were utilized for data cleaning and basic statistical analysis. Since the measured continuous variables did not follow a normal distribution, they were expressed as medians with the interquartile range (IQR). To determine whether the variablesʾ distributions adhered to a normal distribution, evaluations of histograms and the Shapiro-Wilk test were conducted. The differences between the two groups were calculated using U Mann–Whitney tests, whereas between more than 2 groups using the Kruskal-Wallis test. Categorical variables were compared using the χ 2 test or Fisherʾ exact test.

Both survival analyses and visualizations were performed using Python 3.11.5 and its libraries: lifelines 0.27.8, matplotlib 3.7.2, pandas 2.0.3. The Kaplan–Meier estimator was used to estimate both cancer-free survival (time between KTx and cancer diagnosis) and survival after cancer diagnosis (time between cancer diagnosis and all-cause death). Based on the estimator, the estimated median time to either cancer diagnosis or death were calculated. Comparison of survival function between groups was conducted using log-rank test; additional analyses of log(-log) transformed Kaplan-Meier curves were conducted at fixed time points: 1, 3, 5, 10 years. Next, to verify the independency of identified factors, Cox’s proportional hazard model were fitted. In adjusted multivariable models, age (and, to account for non-linear relationship with hazard function, square of the difference between the age of the patient and the mean age of the group).

## Results

The cohort of 3242 KTRs screened towards the inclusion and exclusion criteria, and a total of 246 (7.6%) patients met the eligibility criteria and were included in the analysis. Among these, 140 patients (56.9%) were transplanted in the TC2. Detailed demographic and clinical characteristics of the study group are presented in **Table 1**. The mean follow-up period was 35.7 months (mo) (range 0-230)/2.9y. The longest mean follow-up time was for: testis cancer (53.3 mo/4.4y), kidney cancer (48.1 mo/4y), and colon cancer (46.5 mo/3,8y). The shortest follow-up time was for: biliary tract cancer (4.5 mo/0,37y), lung cancer (9.5 mo/0,8y), and pancreas cancer (12.2 mo/1y).

**Table 1:** Detailed demographic and clinical characteristics of the study group.

	All	Females	Males	*p* value
N	246	88 (35.8)	158 (64.2)	
Age of recipients, median (IQR), years	53.0 (44.0-60.0)	51.0 (40.8-59.0)	53.0 (45.0-60.0)	0.12
Cause of ESKD				0.025^a^
GN, n (%)	98 (39.8)	26 (29.6)	72 (45.6)	
DM, n (%)	20 (8.2)	8 (9.1)	12 (7.6)	
HA, n (%)	17 (6.9)	6 (6.8)	11 (6.9)	
ADPKD, n (%)	34 (13.8)	20 (22.7)	14 (8.9)	
Interstitial nephritis	22 (8.9)	10 (11.4)	12 (7.6)	
Other or unknown etiology, n (%)	55 (22.4)	18 (20.5)	37 (23.4)	
Time of RRT, median (IQR), months	26.0 (15.0-41.0)	24.0 (14.5-41.0)	26.5 (15.0-41.0)	0.69
No data, n	11	5	6	
RRT				0.38
HD, n (%)	206 (86.2)	69 (82.1)	137 (88.4)	
PD, n (%)	24 (10.0)	11 (13.1)	13 (8.4)	
HD+PD, n (%)	3 (1.3)	2 (2.4)	1 (0.6)	
Preemptive, n (%)	6 (2.5)	2 (2.4)	4 (2.6)	
No data	7	4	3	
Number of KTx				0.06
First KTx, n (%)	231 (93.9)	86 (97.7)	145 (91.8)	
Second KTx, n (%)	15 (6.1)	2 (2.3)	13 (8.2)	
Cancer before KTx, n(%)	14 (5.8)	7 (8.3)	7 (4.5)	0.25
No data	5	4	1	
IS before KTx, n (%)	36 (14.6)	12 (13.6)	24 (15.2)	0.74
Induction				0.33
Basiliximab, n (%)	28 (11.4)	12 (13.6)	16 (10.2)	
ATG or thymoglobulin, n (%)	18 (7.4)	8 (9.1)	10 (6.4)	
OKT13, n (%)	1 (0.4)	1 (1.1)	0 (0.0)	
Lack of induction, n (%)	198 (80.8)	67 (76.1)	131 (83.4)	
No data, n	1	0	1	
IS after KTx:				0.73
GC+MMF+TAC, n (%)	94 (38.5)	35 (39.7)	59 (37.8)	
GC+MMF+CsA, n (%)	60 (24.6)	22 (25.0)	38 (24.3)	
GC+AZA+CsA, n (%)	60 (24.6)	17 (19.3)	43 (27.6)	
GC+CsA, n (%)	5 (2.1)	2 (2.3)	3 (1.9)	
GC+TAC, n (%)	4 (1.6)	2 (2.3)	2 (1.3)	
MMF+TAC, n (%)	4 (1.6)	2 (2.3)	2 (1.3)	
Other IS, n (%)	17 (7.0)	8 (9.1)	9 (5.8)	
No data, n	2	0	2	

Groups were compared using Fisher’s exact test, Pearson’s χ^2^ test, and Wilcoxon rank sum test.

A, Pearson’s χ^2^ test;: ADPKD, autosomal dominant polycystic kidney disease; ATG, anti-human thymocyte globulin; AZA, azathioprine; CsA, cyclosporine A; DM, diabetes mellitus; ESRD, end-stage renal disease; GS, glucocorticosteroids; GN, gromerulonephritis; HD, hemodialysis; HA, Hypertension; IS, immunosupressive; KTx, kidney transplantation; MMF, mycophenolate mofetil or mycophenolate sodium; OKT3, anti-CD3 monoclonal antibody; PD, peritoneal dialysis; RRT, renal replacement therapy; TAC, tacrolimus.

### Characteristics of recipients with malignancies

Most of the patients included in the study were male (n = 158; 64.2%). Apart from a slightly different distribution in the causes of kidney failure (p = 0.025), no other significant differences based on sex were observed.

The vast majority of included patients underwent their first kidney transplant (93.9%), while 6.1% the second KTx. Preemptive KTx was performed in 6 patients of our study population.

### Cancer in the pre-transplantation history

In the pre-transplantation period, 5.8% of recipients included in the study suffered from cancer; these were as follows breast cancer (n=3), endometrial cancer (n=1), colon cancer (n=1), kidney cancer (n=2), lymphoproliferative leukemia (n=1), prostate cancer (n=1), head/neck cancer (n=1) and skin cancer (n=4). We observed a recurrence of cancer (breast cancer) after KTx in 3 cases.

### Immunosuppressive medications and previous exposure to IS

14.6% of recipients had a pre-transplant history of IS treatment due to glomerulonephritis (GN) or the first KTx. In the peritransplantation period, 19.1% of recipients received induction therapy, predominantly using basiliximab (11.4%) and anti-human thymocyte globulin (ATG) (7.4%). The most common primary IS regimen consisted of a triple-drug combination, comprising calcineurin inhibitors (CNIs) [tacrolimus (TAC) or cyclosporine (CsA)], an antimetabolite such as mycophenolate mofetil (MMF) or azathioprine (AZA) and glucocorticosteroids (GS). Double-drug combinations were less frequently utilized.

### Cases of cancers observed during the follow-up

A total of 261 malignant tumors were diagnosed in 246 kidney transplant recipients (KTRs). Among these, 3 tumors were recurrences, while the remaining cases were *de novo* occurrences. In 10 recipients, developed two different cancers (native kidney - lung, colon - native kidney, lung - prostatic gland, melanoma - prostatic gland, native kidney - PTLD, prostatic gland - bladder, thyroid-melanoma, native kidney - bile ducts) and one recipient with myeloproliferative disease developed acute leukemia.

In 2 patients three different cancers were noticed (prostatic gland - native kidney - colon, native kidney – lung- adrenal gland).

Interestingly, during the study period, among recipients who developed solid organ cancer, 7.3% also simultaneously developed NMSC.

The cases of each cancer type within the study population, stratified by sex, is detailed in **Table 2**. Furthermore, the analysis revealed a significantly higher proportion of native kidney cancer in males (p = 0.04), while central nervous system (CNS) cancers predominated in females (p = 0.04).

**Table 2:** The cases of each cancer type within the study population, stratified by sex.

Cancer type	ALL n	Cases %	M n	Cases %	F n	Cases %	P
Lung	35	13.4	27	15.7	8	8.9	0.17
Native kidney	34	13.02	28	16.4	6	6.7	**0.04**
Colon	27	10.34	14	8.2	13	14.5	0.11
PTLD	26	9.97	18	10.55	8	8.9	0.67
Prostate gland	24	9.19	24	14	–	–	–
Breast **(3 recurrence)**	16	6.13	0	0	16	17.8	<0.0001 (Fi)
Others (eye, sarcoma, adrenal gland, small intestine, duodenum, vulva)	16	6.13	9	5.3	7	7.8	0.42
Head&neck (nasopharynx, larynx, tongue, salivary gland, thyroid gland)	15	5.74	12	7.1	3	3.3	0.22
Pancreas	11	4.21	7	4.1	4	4.45	1.00 (Fi)
Bladder	9	3.44	7	4.1	2	2.2	0.72 (Fi)
Hematological (besides PTLD)	7	2.68	5	2.9	2	2.2	1.00 (Fi)
HCC	7	2.68	4	2.3	3	3.3	0.69 (Fi)
Melanoma	6	2.29	5	2.9	1	1.1	0.66 (Fi)
Transplant kidney	5	1.91	4	2.3	1	1.1	0.66 (Fi)
CNS	5	1.91	1	0.6	4	4.45	**0.04 (Fi)**
Uterus	5	1.91	–	–	5	5.55	–
Stomach	4	1.53	1	0.6	3	3.3	0.11 (Fi)
Ovary	4	1.53	–	–	4	4.45	–
Testis	3	1.15	3	1.75	–	–	–
Bile ducts	2	0.76	2	1.2	0	0	0.54 (Fi)
**TOTAL:**	**261**		**171**		**90**		

Groups were compared using Fisher’s exact test, Pearson’s χ^2^.

F, females; Fi, Fisher’s exact test; HCC, hepatocellular carcinoma; M, males; PTLD, post-transplant lymphoproliferative disorder. Bold values provided means statistically significant.

**Figure 1:**
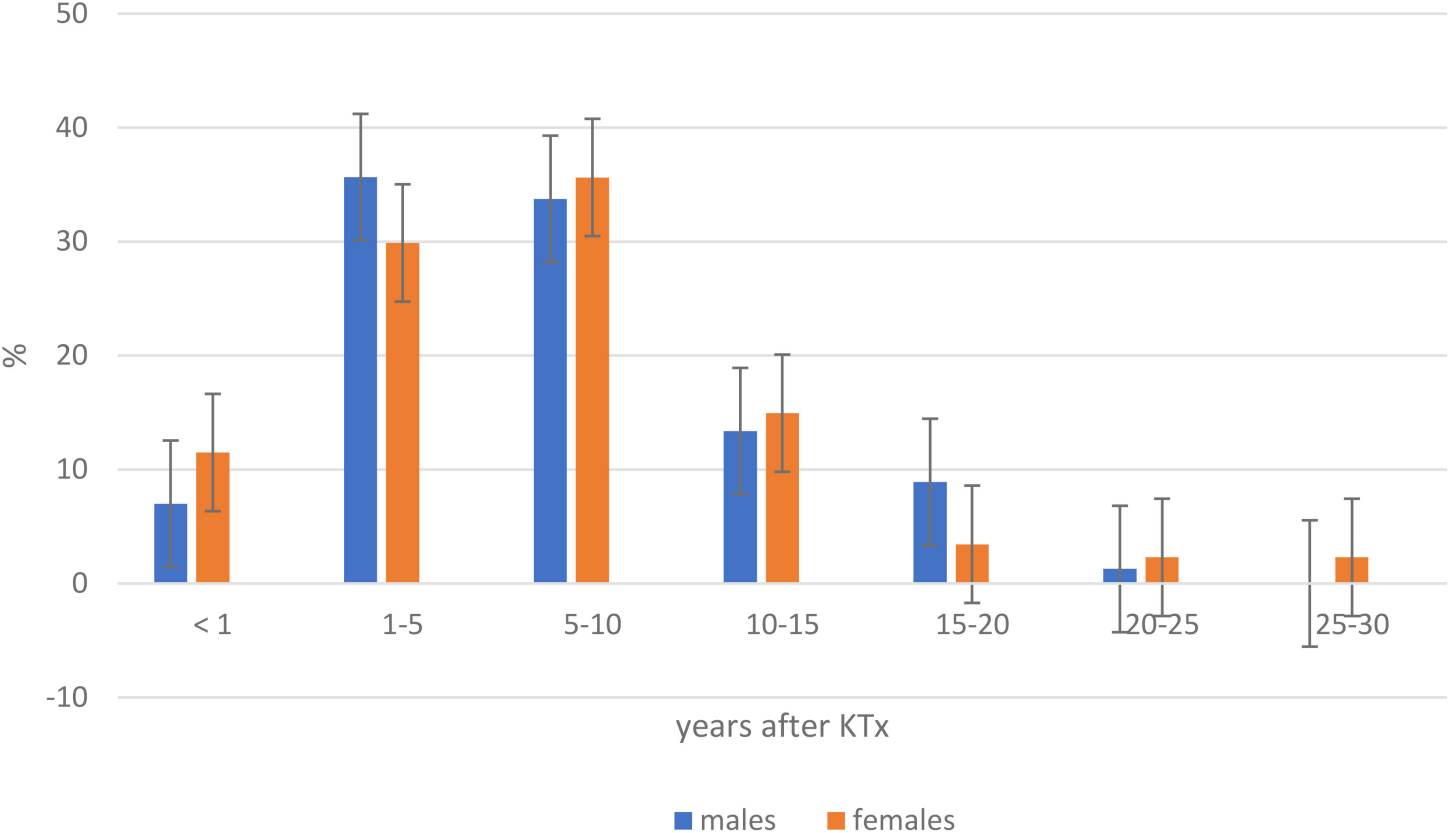
Incidence of cancer after kidney transplantation in time intervals, divided by sex. KTx, kidney transplantation.

The mean age of the recipient at the time of cancer development was 57.8 years. The mean time from transplantation to cancer diagnosis was 84.5 mo/7y. Most cancers were diagnosed within the time frame of 1–5 years (33.60%) and 5–10 years (34.42%) after KTx. Early cancers (<1 year after KTx) were diagnosed more often in females than in males (13.6% vs 8.9%). Late cancers (15–20 years) were more often in males as compare to females (8.91% vs 3.44%). The cases of cancers in time intervals, divided by sex, shows **Figure 1**.

34 kidney cancers (28 males vs 6 females) were diagnosed in native kidneys (p= 0.043), 76.95 mo/6.4y after Tx and 5 in the transplanted kidney (4 male vs 1 female) (p=0.662), 162.6 mo/13.5y after Tx. **Table 3** presents the time of particular cancer diagnosis since KTx.

**Table 3:** Time from kidney transplantation to cancer diagnosis, survival from cancer diagnosis and survival from kidney transplantation.

Cancer	n	Time from KTx to cancer diagnosis Median (IQR) [mo]	Survival from cancer diagnosis Median (95% CI) [mo]	Survival from KTx Median (95% CI) [mo]
Lung	34	62.5 (36.5-100)	7 (3-9)	64 (48-105)
Native kidney	31	55 (35-94.5)	75 (>35)	176 (111-228)
Colon	26	90.5 (48.5-133)	69 (>29)	264 (>150)
PTLD	25	69 (20.3-120)	11 (>3)	121 (72-332)
Prostate	22	92 (55-176)	124 (>40)	242 (>207)
Breast	16	90 (22.5-117.3)	78 (8-227)	169 (107-241)
Head&neck	15	60 (30.5-131.5)	33 (>5)	114 (>74)
Pancreas	11	94 (64-96)	6 (2-20)	98 (53-146)

KTx, kidney transplantation; MO, months; PTLD, post-transplant lymphoproliferative disorder.

#### Cancers in females

The most common cancers after KTx in women were breast cancer (17.8%), colon cancer (14.5%), PTLD, and lung cancer (8.9%) (**Table 2**). Melanoma, PTLD, and CNS cancer occurred in younger recipients (32.0, 45.7, and 45.7 years, respectively). In contrast, bladder, pancreas, and lung cancers developed in older recipients (67.5, 65.0, and 62.6 years, respectively). The earliest diagnosed cancers after KTx were vulva (mean time 39.7 mo/3.3y) and sarcoma (mean time 20 months/1.6y) and the latest after KTx were cancers of the digestive system: hepatocellular carcinoma (HCC) (mean time 143.6 mo/11.9 y), stomach cancer (mean time 118.6 mo/9.9y) and ovarian cancer (mean time 117 mo/9.75y). One case of kidney cancer was diagnosed in transplanted kidneys 246 mo/20.5y after KTx and 6 were diagnosed in native kidneys 74.7 mo/6.2y after Tx. All patients suffering from pancreatic cancer, HCC, and head and neck cancers died due to cancer. None of the female recipients with native kidney cancer and transplanted kidney cancer died due to cancer (1 died due to stroke and 1 due to COVID-19), during follow up 38.8 mo/3.2y. The occurrence of cancer in female - recipients is presented in **Table 4**.

**Table 4:** The occurrence of cancer after kidney transplantation in females.

Cancer type	F N	Cases %	Age at KTx, mean (range), y	Age at cancer, mean (range), y	Time: KTx to cancer, mean (range), mo	Deaths, N (%)	Time: diagnosis to death, mean, mo
Breast (3 recurrence case)	16	17.8	50.5 (31-73)	57.1 (39-80)	73.8 (5-130)	7 (43.75)	59.7
Colon	13	14.5	48.3 (20-74)	55.2 (35-78)	85.2 (24-190)	7 (53.8)	25.7
PTLD	8	8.9	41.4 (20-62)	45.7 (21-65)	75.6 (1-329)	5 (62.5)	7.4
Lung	8	8.9	55 (44-73)	62.6 (44-78)	96.4 (6-219)	7 (87.5)	6.1
Others^a^	7	7.8	47.1 (27-65)	50.6 (29-71)	42.7 (7-76)	3 (42.85)	13.3
Native kidney	6	6.7	54 (31-67)	58.2 (36-75)	74.7 (35-127)	0 (0)	0
Uterus	5	5.55	47 (21-71)	52.6 (27-73)	57.2 (11-157)	3 (60)	25
Ovary	4	4.45	38 (21-55)	48 (38-57)	117 (11-250)	1 (25)	5
CNS	4	4.45	39.25 (30-44)	45.75 (31-53)	79.25 (12-112)	1 (25)	5
Pancreas	4	4.45	58 (50-66)	65 (57-73)	85.5 (7-146)	4 (100)	2.8
Head&Neck (nasopharynx, larynx)	3	3.3	53.3 (43-64)	58.3 (51-67)	60.3 (34-97)	3 (100)	14
HCC	3	3.3	41 (28-65)	52.3 (37-65)	143.7 (3-335)	3 (100)	2.3
Stomach	3	3.3	51.6 (45-59)	61.3 (59-66)	118.6 (173-183)	2 (66.6)	3.5
Bladder	2	2.2	59 (49-69)	67.5 (62-73)	101.5 (47-156)	1 (50)	9
Hematological	2	2.2	57 (55-59)	61.5 (60-63)	61 (59-63)	1 (50)	1
Transplant kidney	1	1.1	26	47	246	0(0)	–
Melanoma	1	1.1	22	32	120	0(0)	–
Total	90						

CNS, central nervous system; HCC, hepatocellular carcinoma; F, females; Mo, months; PTLD, post-transplant lymphoproliferative disorder; Y, years; ^a^ Others (sarcoma, small intestine, duodenum, vulva).

#### Cancers in males

The most common cancers after KTx in males were cancers of the native kidney (16.4%) and cancer of the lung (15.7%), prostatic gland (14%), and PTLD (10.55%) (**Table 2**). Cancers of testis, PTLD, and HCC occurred in younger recipients (44, 50, and 52 years, respectively). In contrast, pancreas, bladder, and prostatic gland cancers developed in older recipients (67, 65, and 65 years, respectively). The earliest after KTx was CNS neoplasm (mean time 26 mo/2.16y), hematological (49.2 mo/4.1y), and testis cancers (56.7 mo/4.7y). The latest after KTx were: HCC (mean time 167.75 mo/13.9y), kidney transplant (mean time 141.75 mo/11,8y), and colon (mean time 121.4 mo/10.1y) cancers. 100% of patients died of stomach, bile ducts and CNS cancer.

There were no deaths due to cancer development in the transplanted kidney. The occurrence of cancer in male recipients is presented in **Table 5**.

**Table 5:** The occurrence of cancer after kidney transplantation in males.

Cancer type	M N	Cases %	Age at KTx, mean (range), y	Age at cancer, mean (range), y	Time: KTx to cancer, mean (range), mo	Deaths, N (%)	Time: diagnosis to death, mean, mo
Native kidney	28	16.4	53.85 (26-70)	60 (34-76)	77.5 (4-193)	6 (22.2)	28.4
Lung	27	15.7	54.85 (32-68)	60 (36-74)	62 (12-146)	23 (85.2)	8.3
Prostate gland	24	14	57.2 (39-76)	64.95 (44-80)	102.9 (9-232)	3 (12.5)	16.5
PTLD	18	10.55	42.2 (18-60)	50.4 (18-60)	86.3 (6 -204)	11 (61.1)	8.5
Colon	14	8.2	48.5 (37-67)	58.6 (34-76)	121.4 (13-263)	2 (14.3)	157
Head/neck^a^	12	7.1	54.25 (41-68)	61.9 (46-74)	93.7 (5-204)	3 (25)	9.4
Others ^b^	9	5.3	49.7 (30-62)	55.1 (37-63)	68.2 (11-128)	4 (44.5)	10.5
Bladder	7	4.1	56.6 (45-60)	65.2 (48-79)	99.15 (26-252)	5 (71.4)	11
Pancreas	7	4.1	59.85 (51-75)	66.85 (56-79)	82.3 (50-126)	6 (85.7)	17
Hematological	5	2.9	53 (31-64)	59 (40-67)	49.2 (7-115)	1 (25)	4
Melanoma	5	2.9	56 (53-64)	62.2 (54-67)	61.5 (18-120)	1 (20)	33
Transplant kidney	4	2.3	40,75 (21-50)	52.5 (43-64)	141.75 (26-276)	0 (0)	–
HCC	4	2.3	37.5 (20-46)	51.75 (39-58)	167.75 (90-224)	2 (50)	1.5
Testis	3	1.75	40 (22-54)	44.3 (24-62)	56.7 (35-100)	1 (33.33)	125
Bile ducts	2	1.2	56 (45-67)	57 (57)	83 (83)	2 (100)	4.5
Stomach	1	0.6	61 (61)	66 (66)	76 (76)	1 (100)	<1
CNS	1	0.6	57 (57)	59 (59)	26 (26)	1 (100)	2
Total:	171						

CNS, Central nervous system; HCC, hepatocellular carcinoma; M, males; Mo, months; PTLD, post-transplant lymphoproliferative disorder; y, years; ^a^ Head/neck (nasopharynx, larynx, tongue, salivary gland, thyroid gland); ^b^ Others (eye, sarcoma, adrenal gland, small intestine).

### The relationship between immunosuppressive protocol and cancers

Pre-transplantation usage of IS was not associated with earlier development of cancer.

In our study group, an analysis of the relationship between the IS used (the three most common regimens) and the time to cancer development was performed.

**Figure 2:**
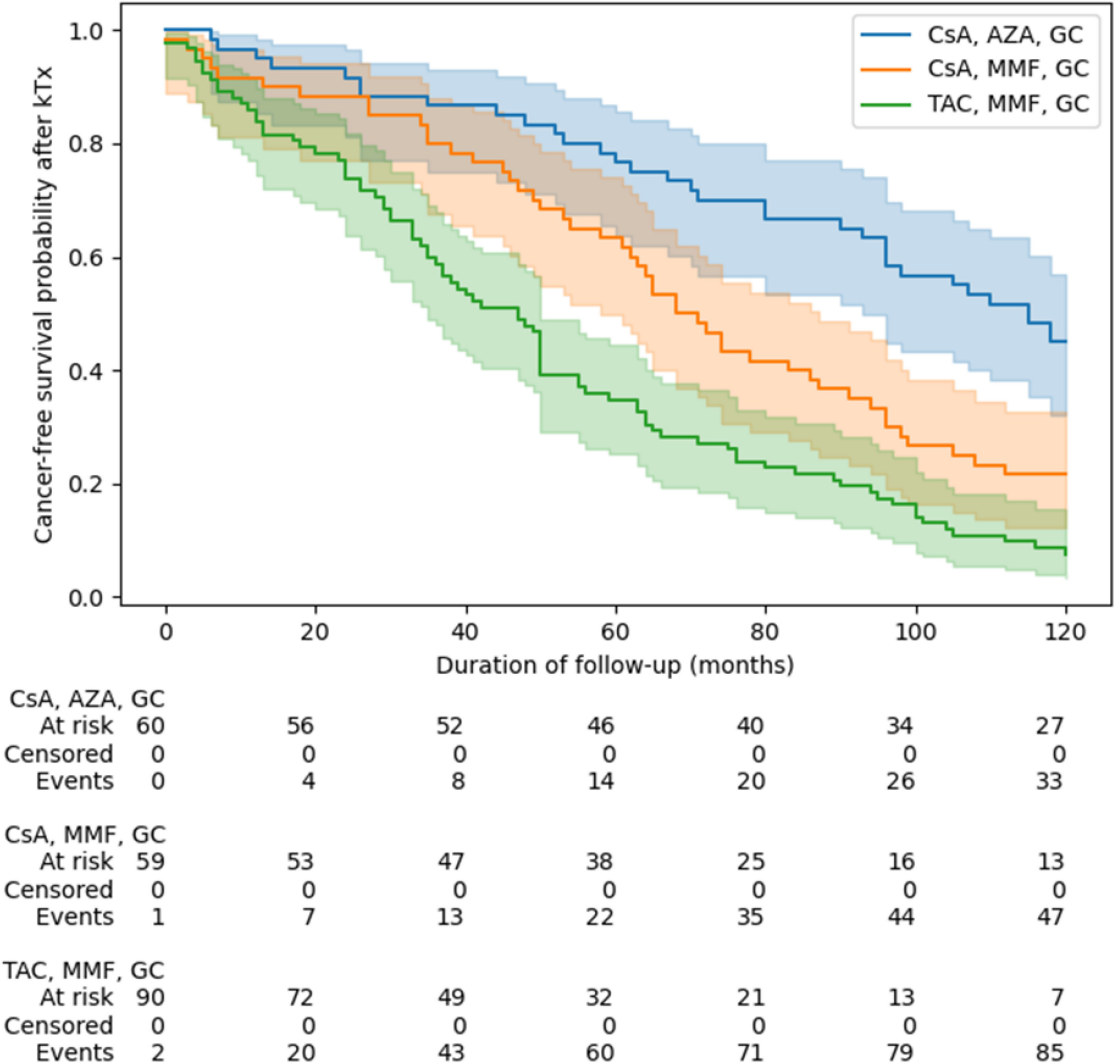
The recipient’s survival from kidney transplantation to cancer diagnosis depending on immunosuppressive protocol. Groups were compared using of log(-log) transformed Kaplan-Meier curves at pre-specified follow-up points (1, 3, 5, 10 years after KTx). AZA, azathiopryne; CsA, cyclosporine A; GS, glucocorticosteroids; MMF, mycophenolate mofetil or mycophenolate sodium; TAC, tacrolimus.

It revealed that the IS regimen was associated with a significant difference in cancer-free survival probability at 3, 5, and 10 years post-KTx follow-up, but not at a short 1-year follow-up (all p > 0.05) (**Figure 2**). Specifically, patients treated with TAC+MMF+GS had a significantly higher probability to be diagnosed with cancer when compared to other regimens at 3, 5, 10 years post-KTx follow-up, while patients treated with CsA+MMF+GC had higher probability when compared with those treated with CsA+AZA+GC only at 10-year follow-up (p = 0.01).

**Figure 3:**
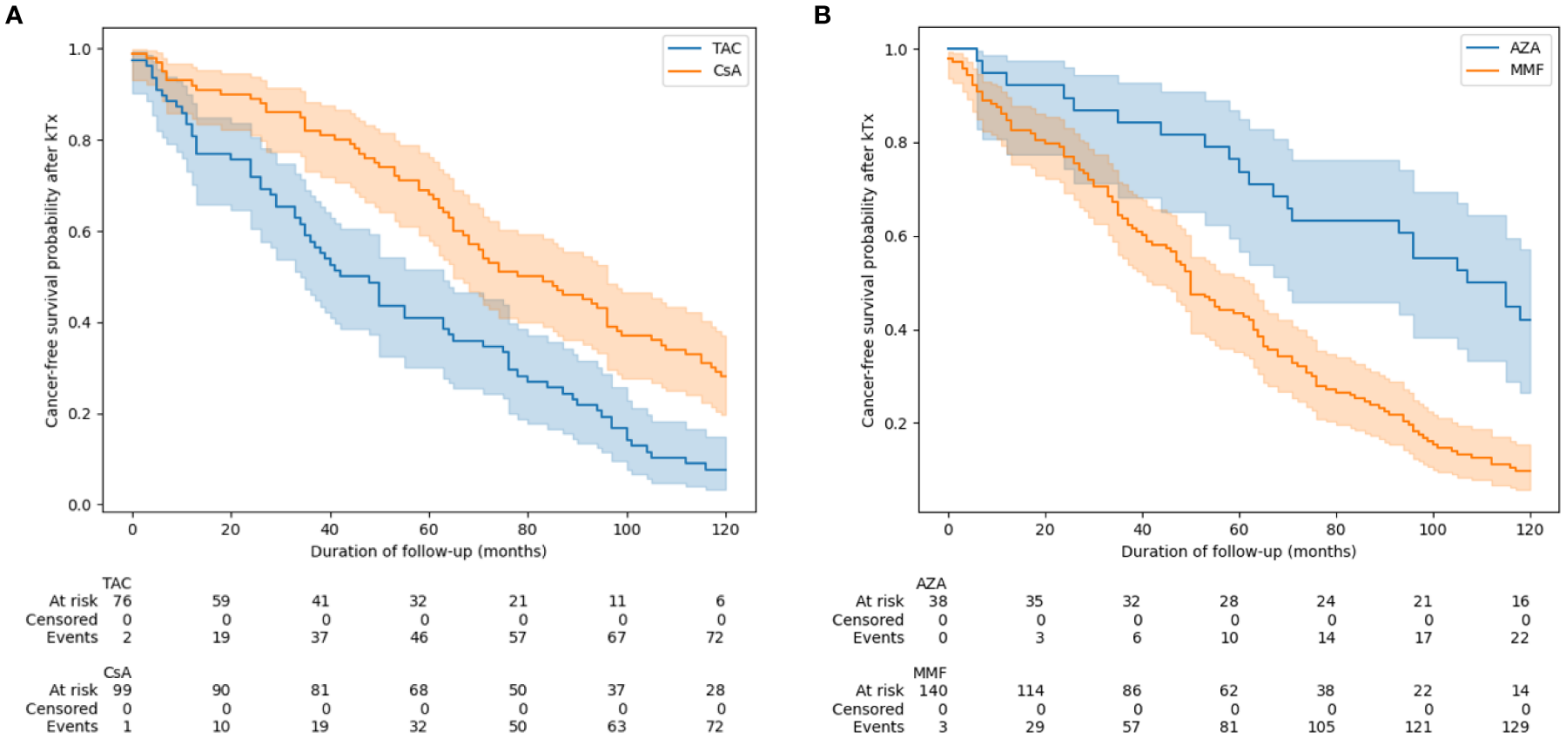
Time to develop cancer depending on the choice of calcineurin inhibitor **(A)** and antiproliferative drugs **(B)**. Groups were compared using of log(-log) transformed Kaplan-Meier curves at pre-specified follow-up points (1, 3, 5, 10 years after KTx). AZA, azathiopryne; CsA, cyclosporine A; MMF, mycophenolate mofetil or mycophenolate sodium; TAC, tacrolimus.

Moreover, CNIs (TAC versus CsA) and antiproliferative drugs (MMF versus AZA) were compared, and the unfavorable effect of TAC and MMF on the development of cancer was also confirmed (**Figure 3**).

**Figure 4:**
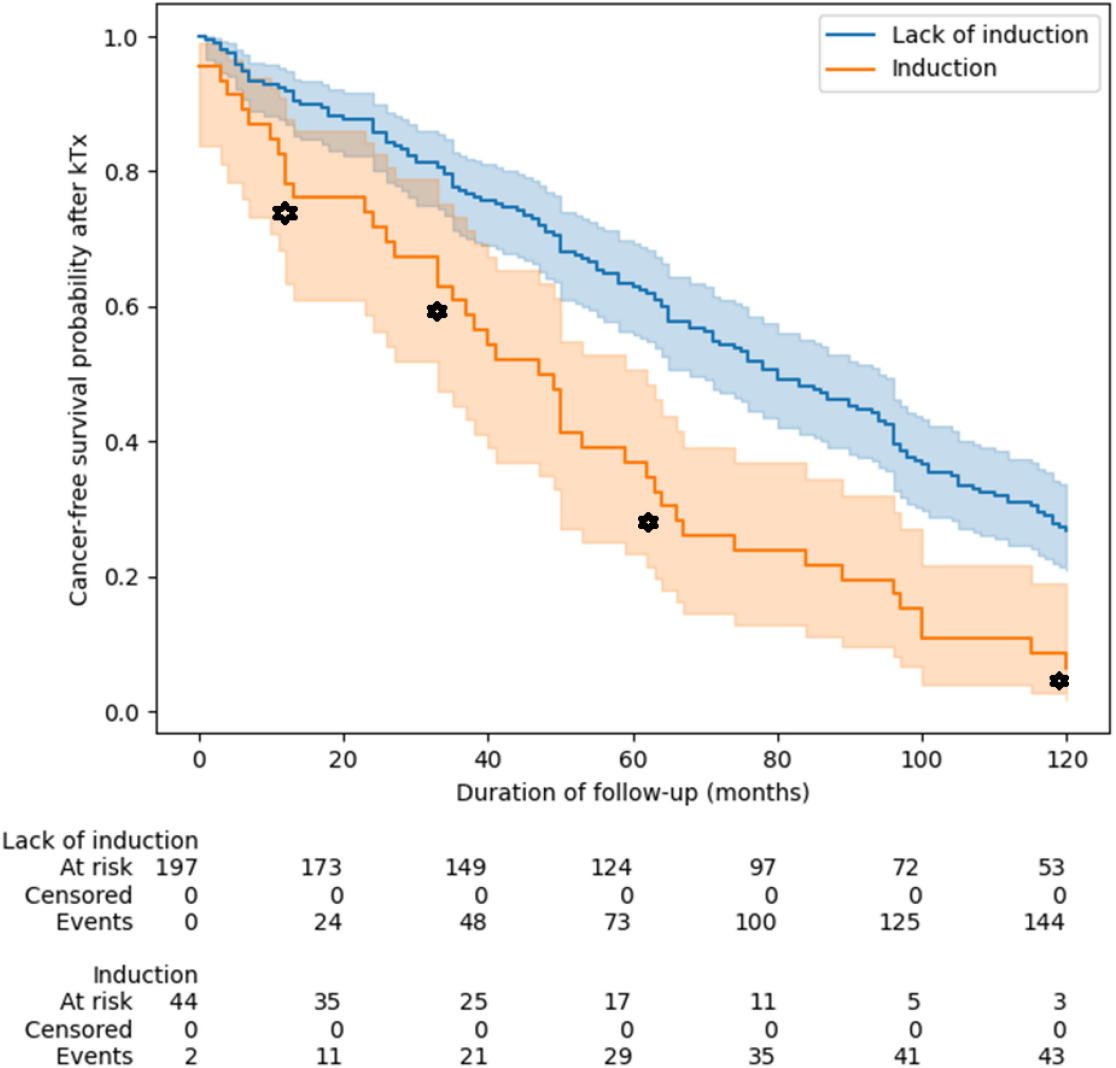
Relationship between induction immunosuppressive therapy and cancer-free survival probability after kidney transplantation. Patients undergoing induction developed cancer earlier compared to patients not undergoing such treatment (log rank test, p < 0.001). p-values ​​for the following points: 1 year: p = 0.009, 3 years: p = 0.02, 5 years: p < 0.001, 10 years: p = 0.001. Groups were compared using of log(-log) transformed Kaplan-Meier curves. KTx, kidney transplantation.

Additionally, patients undergoing induction developed cancer earlier compared to patients not undergoing such treatment (**Figure 4**).

This is also confirmed by multivariable analysis, that patients who received induction (p = 0.02), MMF vs AZA (p= 0.02), or tacrolimus vs CsA (p<0.005) developed cancer earlier after transplantation (**Table 6**). Moreover, the earliest cancers developed in the TAC+MMF+GC group at a median of 47 mo/3.9y) (95% Cl: 35-50), and the latest in CsA+AZA+GC group at a median of 115 mo/9.5y (95% Cl: 93-136).

**Table 6:** Multivariate analysis, time from KTx to cancer.

Variable	Univariate models		Multivariable model^a^	
	HR (95% CI)	*p*	HR (95% CI)	*p*
Antiproliferative drug				
Azathioprine	Baseline	–	Baseline	–
MMF	2.55 (1.71-3.81)	**<0.001**	1.72 (1.07-2.76)	**0.02**
CNI				
CsA	Baseline	–	Baseline	–
Tacrolimus	2.60 (1.84-3.67)	**<0.001**	1.91 (1.32-2.77)	**<0.001**
Induction therapy	2.24 (1.47-3.41)	**<0.001**	1.74 (1.11-2.72)	**0.02**

a: adjusted additionally for age and square of the difference between participant age and mean age in study group

AZA, azathiopryne; CsA, cyclosporine A; MMF, mycophenolate mofetil or mycophenolate sodium; TAC, tacrolimus. Bold values provided means statistically significant.

Multivariate analysis (log-rank test) confirmed a statistically significant relationship between the groups with different IS regimens and the risk of cancer development [(AZA, CsA, P), (MMF, CsA, P) and (MMF, TAC, P) (p < 0.001)]. The posthoc analysis showed differences as follows: (AZA,CSA, P) vs (MMF, CSA, P) p < 0.001; (AZA, CSA, P) vs (MMF, TAC, P) p < 0.001; (MMF, CSA, P) vs (MMF, TAC, P) p = 0.003.

### Patient’s survival

In the analyzed group of 246 KTR, nearly half (48.8%) of patients died due to progression of cancer disease. The mean age of the recipient at the time of death due to cancer was 59.5 years (range 22–80 years). The mean time from cancer diagnosis to death in females was 18.53 mo/1.5y, in males 14.87 mo/1.23y.

Interestingly, death over 6 months from cancer diagnosis occurred in nearly half of the patients 50.42% (55.31% F vs 47.3% M), which suggests a significant advancement of cancer at the time of diagnosis. **Table 3** presents survival from cancer diagnosis and survival since KTx.

We compared patients who died in the course of cancer (regardless of the type of cancer) and those who were cured, the latter differed from the former in terms of baseline nephropathy (p=0.024) and induction treatment, (p=0.04), significantly.

18 patients (7.4%) died for other than cancer reasons, it is worth mentioning that nearly 40% died due to COVID-19 infection. Eighty (32.7%) survived the follow-up of the study. 27 (11.1%) recipients started renal replacement therapy (RRT).

### Graft survival

**Figure 5:**
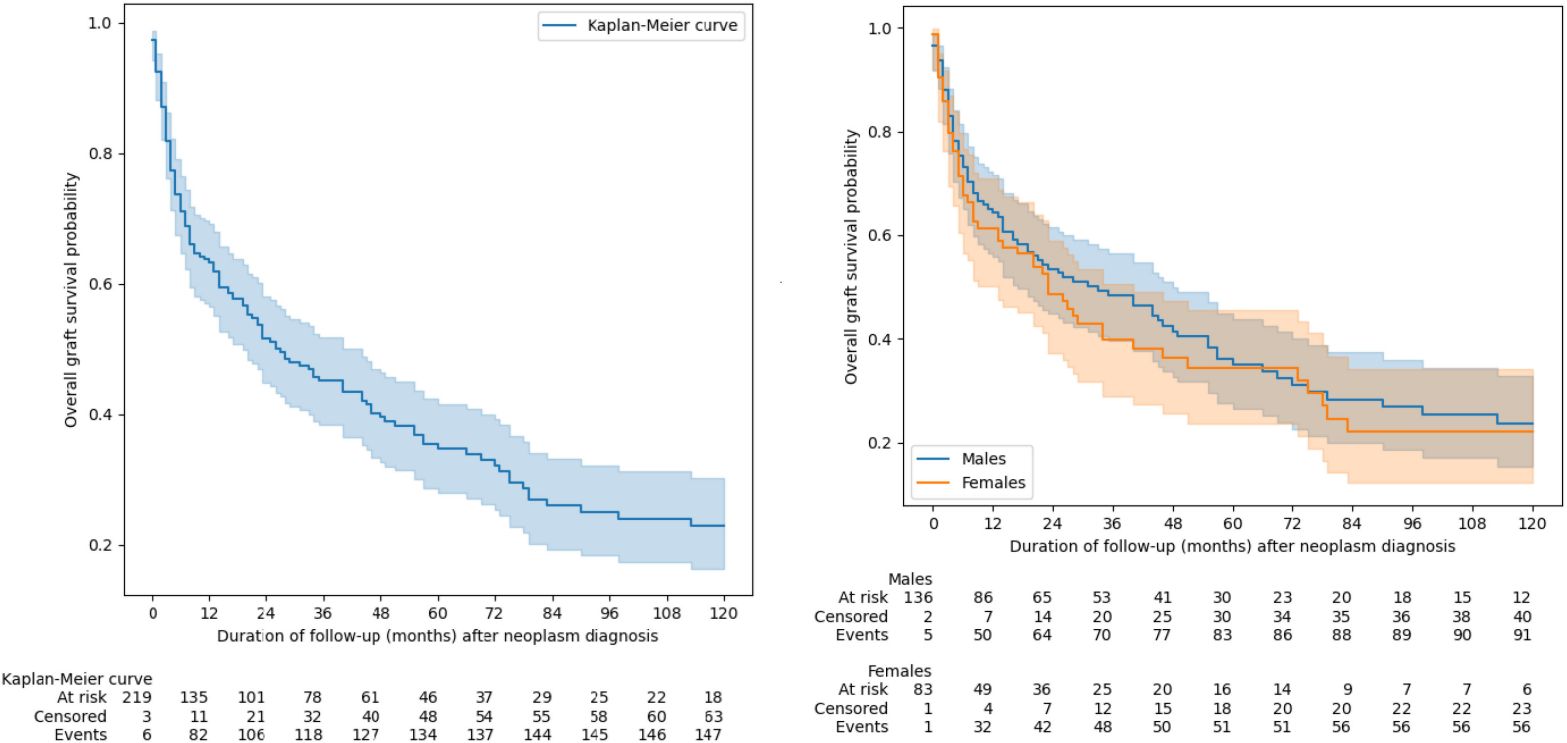
The overall graft survival. Groups were compared using of log(-log) transformed Kaplan-Meier curves.

**Figure 6:**
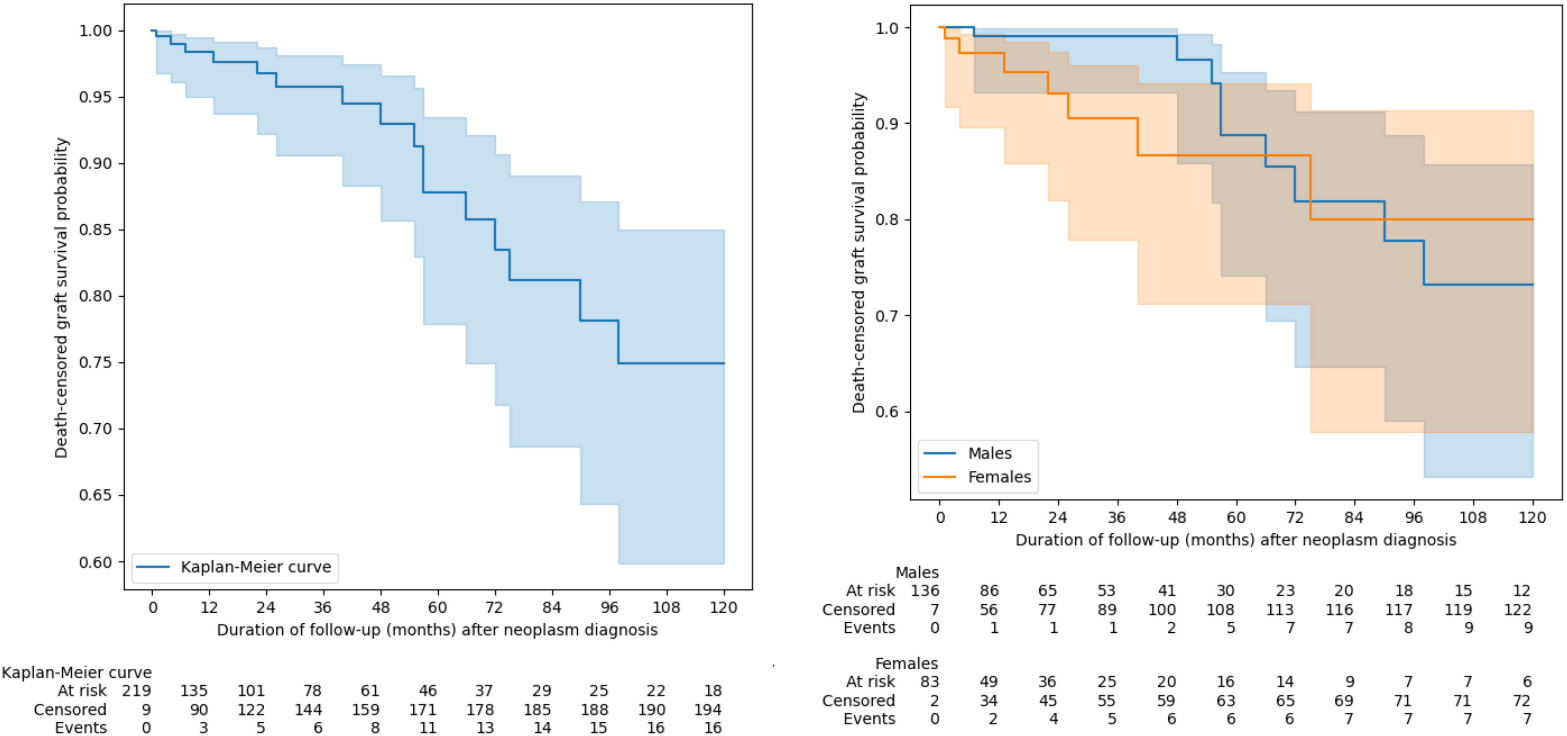
Death-censored graft survival. Groups were compared using of log(-log) transformed Kaplan-Meier curves.

Additionally, we assessed graft survival using Kaplan-Meier curves. In a subgroup analysis of patients who were diagnosed with malignancy while the kidney alllograft was still functioning (patients who had already initiated hemodialysis prior to diagnosis or whom the timing of haemodialysis initiation was unclear were excluded from analysis), neither overall nor death-censored graft survival differ between sexes (**Figures 5**, **6**). The differences between the overall and death-censored graft survival curves were striking, clearly indicating that the primary cause of graft loss in this subgroup was patient death rather than renal allograft failure. Most patients died with a functioning graft, underscoring the impact of mortality - rather than graft dysfunction - on overall graft outcomes in this population.

### The relationship between immunosuppressive protocol and patient’s survival

**Figure 7:**
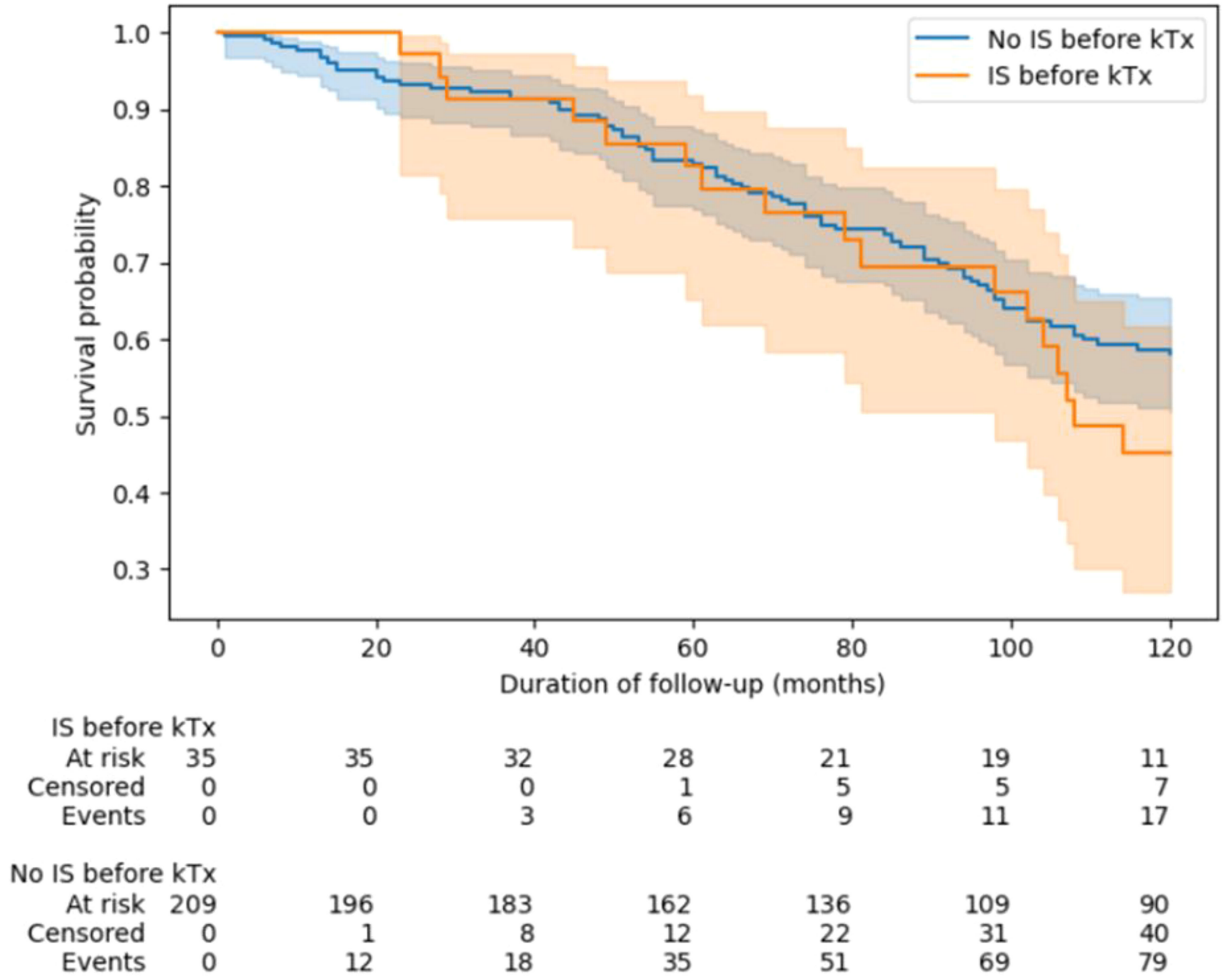
Survival analysis of kidney recipients depending on the use of immunosuppression before transplantation. Groups were compared using of log(-log) transformed Kaplan-Meier curves. IS, immunosupressive; KTx, kidney transplantation.

In statistical analysis, we did not show that the group of recipients treated with IS before Tx had poorer survival after KTx as compare to the rest of the studied patients (**Figure 7**).

In both univariable and multivariable analyses (adjusted for sex and age), we found that induction therapy was associated with an increased risk of death only in patients with lung cancer or PTLD (**Table 7**). In contrast to the risk of cancer development, we did not observe significant differences in the risk of death between patients treated with TAC versus CsA or patients treated with MMF versus AZA.

**Table 7:** Univariable and multivariable analyses immunosuppressive and survival.

Variable	All-cause mortality (univariable analyses)							
	Lung cancer		Native kidney cancer		Colon cancer		PTLD	
	HR (95% CI)	*p*	HR (95% CI)	*p*	HR (95% CI)	*p*	HR (95% CI)	*P*
Antiproliferative drug								
Azathioprine	Baseline	–	–	–	Baseline	–	Baseline	–
MMF	0.54 (0.19-1.52)	0.24	-^c^	–	0.42 (0.07-2.58)	0.35	0.49 (0.15-1.62)	0.24
CNI								
CsA	Baseline	–	Baseline	–	Baseline	–	Baseline	–
Tacrolimus	1.24 (0.53-2.88)	0.62	1.18 (0.19-7.16)	0.86	0.62 (0.12-3.21)	0.57	0.92 (0.28-3.08)	0.89
Induction therapy	4.88 (1.81-13.16)	**0.002**	0.30 (0.04-2.34)	0.25	-^c^	–	7.22 (1.98-26.36)	**0.003**

Variable	All-cause mortality (adjusted for age and sex)							
	Lung cancer		Native kidney cancer		Colon cancer		PTLD	
	HR (95% CI)	*p*	HR (95% CI)	*p*	HR (95% CI)	*p*	HR (95% CI)	*p*
Antiproliferative drug								
Azathioprine	Baseline	–	–	–	Baseline	–	Baseline	–
MMF	0.52 (0.18-1.53)^a^	0.24	-^c^	–	-^c^	–	0.26 (0.06-1.10)	0.07
CNI								
CsA	Baseline	–	Baseline	–	Baseline	–	Baseline	–
Tacrolimus	1.18 (0.49-2.81)^b^	0.71	0.98 (0.15-6.50)^a^	0.99	0.25 (0.02-2.60)^b^	0.25	1.02 (0.28-3.68)	0.98
Induction therapy	5.82 (2.02-16.77)^a^	**0.001**	0.23 (0.03-1.92)^a^	0.18	-^c^	–	6.71 (1.69-26.63)	**0.007**

^a^: adjusted for sex and age; ^b^: adjusted for sex, age, and (age)2 to account for non-linearity; ^c^: model cannot be reliably fitted (95% CI from 0 to infinity). Bold values provided means statistically significant.

## Discussion

Cancer, next to cardiovascular diseases (CV) and infections, is the main cause of death in patients after KTx (6–11). Data based on large databases indicate that the risk of cancer after KTx (except NMSC) is 2–3 times higher than in the general population (7, 9, 10, 12, 13). The risk is even 5–10 times higher, for cancers related to infection with oncogenic viruses such as PTLD and Kaposi’s sarcoma (7, 9).

The incidence of cancers in the general population and KTRs is distributed differently (14). In our cohort, 7.6% of KTRs developed cancers and it was similar to the other studies (7.2%, 6%, 7.6%, 6% respectively) (6, 9, 12, 15). Some literature reports have shown that males suffer from post-KTx cancer more often than females (1, 6, 9, 12, 16). Our results in the group of KTRs are in line with these observations since males constituted the majority of patients suffering from cancer.

In the general population of Poland, the most frequently diagnosed type of cancer in males remains lung cancer, prostate cancer, and colon cancer (18%, 18%, and 15% in the 2020 year, respectively) (2, 3), whereas kidney cancer is responsible for only 3.6% cancer cases (17).

Our results in KTRs revealed that the most common cancer in males was native kidney cancer (16.4%), whereas colorectal cancer was surprisingly less common (8.2%). In females, in the general population of Poland, the most frequently diagnosed cancers remain: breast cancer (25%), lung cancer (12%), and colorectal cancer (11%) in 2020 (2, 3), kidney cancer is responsible for only 2.6% cancer cases (17). In our cohort of female KTRs the most diagnosed cancer was also breast cancer (17.8%), colorectal cancer (14.5%), followed by PTLD and lung cancer (8.9%) and native kidney cancer (6.7%).

Hickman et al. observed that approximately 90% of renal cell carcinoma (RCC) is diagnosed in native kidneys in KTR (18). Our study, confirms that observation, therefore 87% of RCC were diagnosed in native kidneys, and only 13% in the transplanted kidneys. In the study by Gisco et al. (6), native kidney cancer was one of the most frequently diagnosed cancers after KTx. Moreover, Hickman et al. demonstrated even 7 times higher cases of native kidney cancer in KTRs as compared to the general population (18).

The risk of RCC development and urinary tract cancer is not only higher among patients with ESRD, but also it increases with the duration of dialysis and after KTx (19).

An association of dialysis vintage and risk of urinary tract cancers (kidney, urothelial, and bladder) after Tx has also been found by Wong et al. in the ANZDATA registry (7, 20). After KTx, the risk factors for kidney or urinary tract cancer increase due to aforementioned factors and also BKV infections (21). Additionally, in retransplants compared to primary kidney transplants in the study in United States, among all malignancies only RCC was found at a higher incidence (22). Retransplanted recipients had two-fold higher incidence of RCC than primary recipients.

The pathogenesis of RCC in CKD is not fully understood, it is suggested that is associated with renal fibrosis and tubular atrophy, chronic inflammation associated with uremia, oxidative stress, impaired immune function, the dialysis process, medications and comorbidities (7). Increased prevalence of RCC could be also explained by its association witch acquired cystic kidney disease (ACKD), which is a risk factor for RCC (18, 23). ACKD is highly prevalent in patients with CKD. In ACKD, cysts originate in dilated renal tubules, and increase in number over time, even before the need for RRT (19). After initiation of dialysis, the prevalence of cysts continues to increase, with the majority of patients having cysts after 10 years of dialysis, suggesting that the duration of CKD or dialysis is the main risk for development of renal cysts (24). RCCs can arise within complex cysts present in ACKD (25, 26). Prolonged dialysis and development of ACKD may partly explain high risk of RCC in transplant recipients and also those retransplanted.

KDIGO Clinical Practice Guideline on the Evaluation and Management of Candidates for Kidney Transplantation 2020, underline these facts recommending screen with ultrasonography among kidney transplant candidates particularly those possessing following risk factors: ≥ 3 years dialysis, family history of renal cancer, ACKD or analgesic nephropathy and long term smoking. They suggest also urine cytology and cystoscopy to screen for bladder carcinoma in candidates at increased risk (high-level exposure to cyclofosfamide or heavy smoking (30 pack-years) (27).

The histopathological pattern of RCC is different in KTRs (mostly papillary, multifocal RCC) and the general population (renal clear cell carcinoma) (23, 28).

According to KDIGO, no increased rate of RCC was found among ADPKD patients (14). However, a study of removed kidneys from ADPKD showed as high as 5-8% cases of RCC. This observation raises concerns about the malignant potential in ADPKD kidneys.

In our study population, ADPKD was a cause of ESRD in 13.82% of patients. However, only one of them developed kidney cancer in a polycystic kidney.

Kidney cancer can be treated radically and the prognosis is good, in both the general and in the KTRs population (29). In our study, among patients with native kidney RCC, 82.4% are still alive. The majority of kidney cancers were detected accidentally during routine ultrasound examinations, therefore in KTRs ultrasound of the abdomen is recommended in RCC screening (30).

The peak incidence of cancer occurs 3–5 years after Tx (9, 11). In our group of recipients, most cancers occurred in the intervals 1–5 years (33.6%) and 5–10 years (34.42%) after KTx.

The risk of cancer development after KTx is influenced by pre-transplantation risk factors same as in the general population, and risk factors related to the transplantation itself such as posttransplant IS treatment. It should be mentioned, that some patients receiving KTx are already under IS treatment used in the pre-transplantation period, either due to the treatment of disease-causing ESRD or because they were already a recipient of another transplanted organ, which again increases the risk of complications of IS treatment.

In our study, 14.6% of patients receiving IS before KTx developed *de novo* cancers after KTx, but it was not associated with earlier development of cancer and did not contribute to poorer survival after KTx.

That’s well known, that IS drugs impair T-cell function, immune surveillance and immune control (11). TAC increases the level of TGF-B, which promotes cancer development. CsA also has a direct effect on tumor development and progression through the expression of TGF-β or IL-6 (13, 29). According to the literature, TAC increases the risk of lymphoma twice as much as CsA (13). Among older drugs, AZA increases the risk of lymphoma and NMSC (9, 13, 31).

Data from the literature suggest that less intense IS significantly reduces the risk of cancer, although increases the risk of rejection (32).

We showed that the use of induction and the IS regimen with TAC and MMF predisposed significantly to the earlier development of cancer, which is in line with the literature.

The risk of developing cancer after KTx is additionally increased in infection with oncogenic viruses promoted by IS (6–10, 12, 13, 32). For example, the HHV-8 virus increases the risk of developing Kaposi’s sarcoma 20 times (7, 10), and the human papillomavirus (HPV) increases the risk of cervical cancer 5–10 times (7), additionally, HPV is a risk factor for anus, penis, and vagina, oropharyngeal cancers (32) and may also cause genital warts in the urinary bladder with a high malignant potential in immunocompromised patients (21). In our study of cancers there were no data regarding the viral status thus, the coincidence with mentioned viral infection could not have been analyzed.

The cases of PTLD is also increased in the case of Epstein -Barr virus (EBV) infection (7, 33), while the BKV increases the risk of urinary tract cancer, hepatitis C, B virus increases the risk of liver cancer (6, 7, 12, 13, 21).

According to literature data, the EBV is the cause of approximately 60-90% cases of PTLD (9, 11, 29, 33). The use of polyclonal antibodies was associated with consistently higher risk of lymphoma (13). Due to the non-specific symptoms of PTLD, the disease is often diagnosed late (over two years after Tx) (33). The average time from diagnosis of PTLD to death is 6 months according to literature data (34). The incidence of PTLD was associated with administration of the induction therapy and the prognosis of these patients was unfavorable. In our study, PTLD was the fourth most common cancer after KTx. In both univariable and multivariable analyses, we found that induction therapy was associated with an increased risk of death in patients suffering from PTLD.

After developing cancer, the risk of death among KTRs increases (6, 16). Taborelli et al., compared KTRs with and without cancer. The 5-year survival rate among KTRs with cancer (without NMSC) was 63%, and the 5-year survival rate among KTRs without cancer was 89% (16). In another study, 46.2% of KTRs died due to cancer, and our study also showed a similar (48.8%) percentage of deaths (6). In a South Korean study, Jeong et all, showed that mortality associated with cancer after transplant was 14.9% (12). The difference may be because Jeong, unlike us, includes skin cancer, which has a very good prognosis. In our study death over 6 months from cancer diagnosis occurred in nearly half of the patients which suggests a significant advancement of cancer at the time of diagnosis. We compared patients who died in the course of cancer (regardless of the type of cancer) and those who were cured, the latter differed significantly from the former in terms of baseline nephropathy and induction treatment. In both univariable and multivariable analyses, we found that induction therapy was associated with an increased risk of death only in patients with lung cancer or PTLD. In contrast to the risk of cancer development, we did not observe significant differences in the risk of death between patients treated with TAC versus CsA or patients treated with MMF versus AZA.

In the Benoni study, patients with cancer after solid organ transplantation were compared with patients with cancer without transplantation (35). There was a 35% higher death rate among solid organ recipients. A higher mortality rate was observed among patients with lymphoma, melanoma, urothelial cancer, breast, head and neck, and colorectal cancer. There was a similar percentage of deaths between both groups among patients with prostate, lung, and kidney cancer.

According to KDIGO in recipients diagnosed with cancer before KTx, the risk of death due to cancer is three times higher than among recipients without a history of cancer (7).

In another study, Dahle et al. (36) compared KTRs with a history of cancer before transplantation (6.4%) with recipients without a history of cancer. Follow-up lasted 6.8 years, 13.7 died due to cancer recurrence, and 10.5% died due to *de novo* cancer. Regardless of whether the recipient suffered from cancer before or after KTx, when they suffered from cancer, they had similar overall survival (36). The greatest number of people cured were in the group of recipients who suffered from RCC, PTLD, prostate cancer, and colon cancer.

The 5.8% of KTRs in our cohort suffered from cancer before transplantation, in three cases recurrence of the disease was observed. All three recurrences were breast cancer, mean time from transplantation to recurrence was 70 mo/5.8y (range 10-118), two patients died.

Although the exact number of donor- transmitted malignancies is not known, based on the available registries and published data, the risk is approximately 0.05% (37). United Kingdom Transplant registry from 10-year period described the risk of transmission as 0.06% (these included 14–986 donors) (38). Spanish registry (39), based on a data from 1990 to 2006 year assessed the risk of transmission of malignancy to the recipients as 0.06%. Italian registry documented that the risk of transmission was 0.03% (40). Although the risk of transmission is rare but it has serious consequences (41). To ascertain the origin of the neoplasm, immunohistochemistry and molecular analysis can be employed (fluorescence *in situ* hybridization, microsatellite allelic analysis, and comparative genomic hybridization (41).

The presented study has some limitation: 1) the studied group of recipients is medium-sized, 2) in 3.65% of patients, who started RRT or were transferred to another center, we do not know what happened afterwards and 3) statistical analysis regarding death from any cause vs. alive is subject to the risk of systematic error (bias) because there are no people without cancer in the database. 4) Dosages and IS drug levels were not included because most of these data are limited.

## Conclusions and recommendation on the base of literature confirmed additionally by our study

The presented study shows a different distribution of cancer cases in patients after KTx as compare to the general population.

Patients after KTx require regular screening for neoplastic diseases, with particular emphasis on neoplastic diseases of the urinary system. Our findings support the need for RCC screening in all transplant candidates and recipients. This procedure should be obligatory in all candidates before including on waiting list and individualized after KTx. Recommending an annual inspection by ultrasound particularly in patients with suspicious lesions such as ACKD seems reasonable. All those possessing other pretransplant conditions mentioned in KDIGO guidelines should also be checked on individual way.

## Data Availability

The original contributions presented in the study are included in the article/supplementary material. Further inquiries can be directed to the corresponding author/s.
